# Surgical release of a deep retro-equatorial band in congenital unilateral restrictive strabismus: a case report

**DOI:** 10.1097/RC9.0000000000001160

**Published:** 2026-10-22

**Authors:** Abdulrahman Alyahya, Fawzia Alhaimi

**Affiliations:** Pediatric Ophthalmology, Strabismus and Uveitis, King Khaled Eye Specialist Hospital and Research Center, Riyadh, Saudi Arabia

**Keywords:** anomalous orbital tissue, case report, congenital restrictive strabismus, forced duction testing, mechanical restriction, retro-equatorial band

## Abstract

**Introduction and importance:**

Congenital unilateral restrictive strabismus may result from anomalous orbital tissue, extraocular muscle abnormalities, or congenital cranial dysinnervation. Forced duction testing can identify a passive mechanical component, but does not establish tissue identity or exclude coexisting dysinnervation.

**Presentation of the case:**

An 11-year-old girl had congenital ocular misalignment with 40 prism diopters (PD) of right hypotropia, 20 PD of exotropia, and left supraduction and infraduction each graded −3, with full abduction and adduction. Apparent right upper-eyelid asymmetry was formally evaluated by the oculoplastic service and diagnosed as pseudoptosis associated with the right hypotropia rather than true ptosis; margin reflex distance-1 was 3 mm bilaterally with each eye fixating, and levator function was >14 mm bilaterally. Computed tomography showed intact orbital walls without obvious extraocular muscle entrapment; dedicated orbital magnetic resonance imaging was not performed. Under general anesthesia, multidirectional forced duction testing demonstrated no appreciable restriction during passive elevation in adduction. Maximum resistance was localized to a focal central point in the superior plane, while passive infraduction and horizontal rotations were unrestricted. Superior exploration identified a focal tissue attachment at the nasal aspect of the normal-appearing superior rectus region and, more posteriorly, a deep retro-equatorial band. Stepwise release markedly improved passive elevation, although mild residual superior restriction remained because further posterior dissection was limited by the risk to deep orbital neurovascular structures and the optic nerve. No tissue was submitted for histopathological examination. A 10 mm left lateral rectus recession was performed for the associated exotropia. At 12 months, horizontal alignment was orthotropic, and residual right hypotropia measured 12 PD at distance and approximately 8–10 PD at near. Left supraduction and infraduction had both improved to approximately −2, while abduction and adduction remained full.

**Clinical discussion:**

The intraoperative response supports an important mechanical contribution, although the identity, full posterior extent, and anatomical relationships of the abnormal tissue remain uncertain. The documented diagnosis of pseudoptosis with preserved levator function, rather than true congenital ptosis, makes classic congenital fibrosis of the extraocular muscles (CFEOM)1 and CFEOM2 less characteristic; however, CFEOM3 cannot be excluded without dedicated orbital/cranial nerve imaging and, when indicated, genetic assessment. Residual vertical motility limitation may reflect the safely unreleased posterior restriction, coexisting extraocular muscle dysplasia or dysinnervation, or a combination of these factors.

**Conclusion:**

Stepwise partial release of a deep retro-equatorial band substantially reduced the accessible passive restriction while avoiding unsafe posterior dissection. At 12 months, vertical alignment and both supraduction and infraduction had improved, although residual right hypotropia and vertical motility limitation persisted. The findings support an important mechanical component but do not establish the precise tissue identity or exclude coexisting congenital dysinnervation.

HIGHLIGHTS

- Oculoplastic assessment confirmed pseudoptosis rather than true ptosis.
- Serial forced duction testing identified a focal superior mechanical restriction.
- The partial release of a deep retro-equatorial band improved passive elevation.
- Posterior dissection was limited to protect the optic nerve and deep structures.
- At 12 months, vertical alignment and vertical ductions remained improved.

## Introduction

Congenital unilateral restrictive strabismus is uncommon and has a heterogeneous differential diagnosis. Mechanical or anatomical causes include extraocular muscle or tendon dysplasia^[^1,2^]^, anomalous accessory orbital structures^[^2–5^]^, and congenital orbital fibrosis or fibrosis-like abnormalities^[^6–10^]^. Reported anomalous orbital bands vary in origin, attachment, and posterior extent and may produce complex incomitant deviations^[^2–5^]^. Positive forced duction testing demonstrates a passive mechanical component but does not establish the identity of the restricting tissue or exclude coexisting congenital dysinnervation.

Congenital fibrosis of the extraocular muscles (CFEOM) is an important congenital cranial dysinnervation disorder^[^11^]^ characterized by congenital, non-progressive ophthalmoplegia with variable ptosis^[^12–14^]^. Classic CFEOM1 typically presents with bilateral ptosis, infraducted eyes, and marked limitation of elevation, whereas CFEOM2 is generally associated with bilateral ptosis, severe ophthalmoplegia, and exotropia. CFEOM3 has a more variable phenotype and may be unilateral or asymmetric, with ptosis variably present or absent^[^13,14^]^. Consequently, neither unilateral involvement nor identification of a mechanical restriction alone is sufficient to exclude CFEOM3.

Acquired causes, including traumatic entrapment or scarring, orbital inflammation, thyroid-associated orbitopathy, and postoperative adhesions, should also be considered according to the clinical history and examination. We report the surgical identification and partial release of a deep retro-equatorial band in a child with congenital unilateral restrictive strabismus, with emphasis on distinguishing operative anatomy, passive mechanical restriction, and active ocular motility. The abnormal tissue is described anatomically because its histological identity was not established. This report follows the revised SCARE guidelines^[^15^]^.

## Presentation of the case

An 11-year-old girl presented with ocular misalignment that had been noticed by her family since birth. A history of minor trauma in early infancy was reported; however, the ocular deviation had been observed before the traumatic event and was therefore not considered causal. The deviation was long-standing, stable, and non-fluctuating. There was no history of pain, orbital inflammation, thyroid disease, previous ocular surgery, systemic illness, or a family history of strabismus or congenital fibrosis syndromes. Best-corrected visual acuity was 20/20 in each eye, and anterior- and posterior-segment examinations were normal.

Preoperative examinations consistently demonstrated marked right hypotropia. An earlier assessment documented 16 prism diopters (PD) of exotropia with 40 PD of right hypotropia. At the subsequent preoperative assessment, used for surgical planning, exotropia measured 20 PD, while the right hypotropia remained 40 PD. Active motility of the left eye demonstrated limitations in both supraduction and infraduction, each graded −3, with full abduction and adduction. Extraocular motility of the right eye was full. Preoperative nine-gaze photographs demonstrated restricted left elevation in both adduction and abduction (Fig. 1). The patient did not report diplopia or adopt an abnormal head posture.

**Figure 1.**
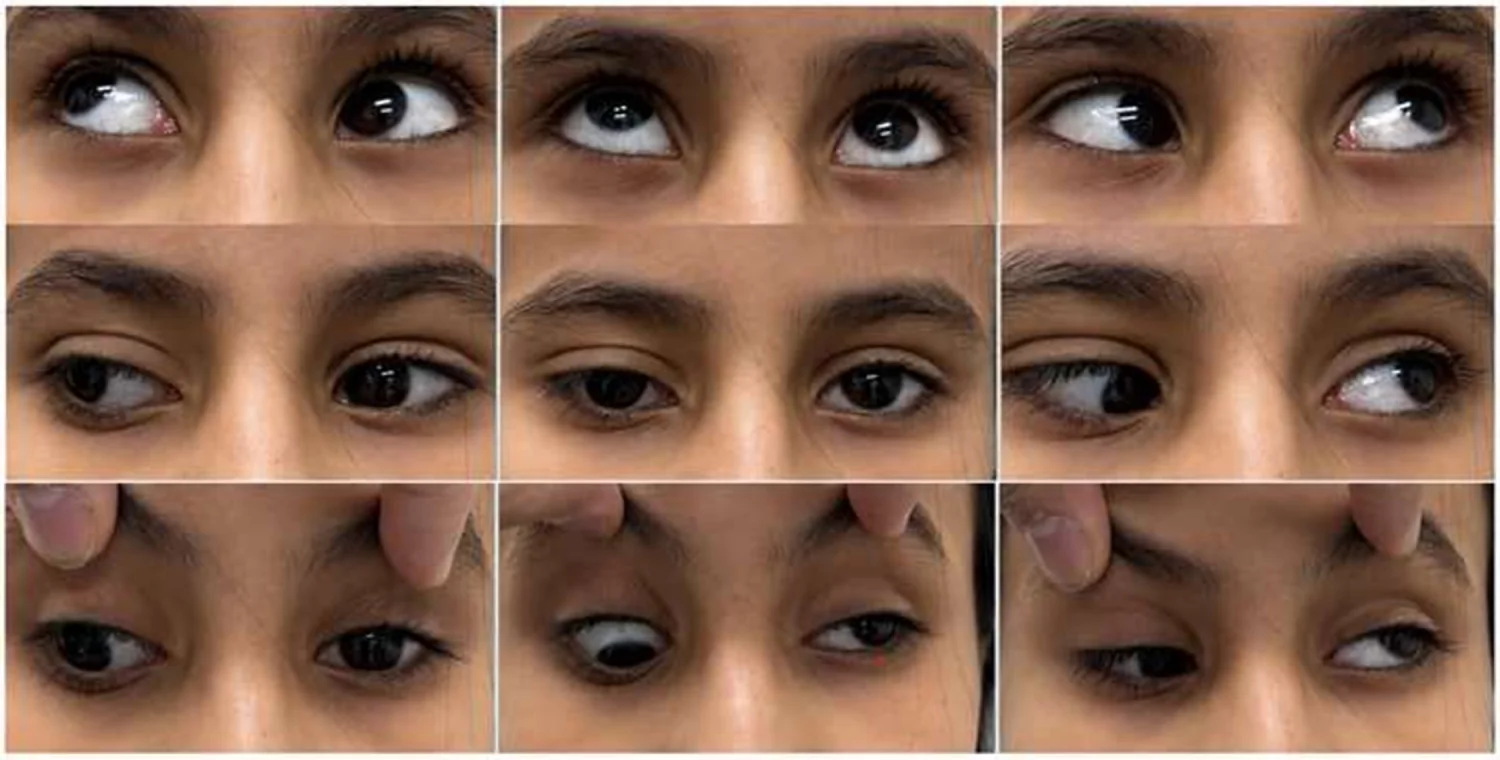
Preoperative nine-gaze ocular-motility photographs. The left eye (viewer’s right) shows hypertropia and exotropia in primary position and a limitation of elevation and depression. Clinical examination documented left supraduction and infraduction, each graded −3; full abduction and adduction; 40 PD right hypotropia; and 20 PD exotropia at the preoperative assessment used for surgical planning. Right upper-eyelid asymmetry is visible; a separate oculoplastic evaluation diagnosed pseudoptosis associated with the right hypotropia rather than true ptosis. The upper eyelids were gently elevated in the downgaze row to improve globe visualization.

Right upper-eyelid asymmetry was assessed separately by the oculoplastic service and was diagnosed as right pseudoptosis associated with right hypotropia, rather than primary ptosis. The oculoplastic assessment documented a margin reflex distance-1 (MRD-1) of 3 mm in both eyes when each eye was fixating, palpebral fissure measurements of 9–10 mm, and levator function greater than 14 mm bilaterally. Upper-eyelid creases were formed bilaterally, with no lid lag in downgaze and no lagophthalmos; Bell’s phenomenon was good.

Orbital computed tomography (CT) was obtained because of the remote history of trauma. The orbital walls were intact, with no obvious extraocular muscle incarceration or other traumatic orbital abnormality. Because the ocular deviation had been present since birth and had preceded the trauma, an acquired traumatic cause was considered unlikely. However, CT could not exclude a subtle extraocular muscle or tendon, accessory soft-tissue, or deep orbital abnormality. Dedicated orbital magnetic resonance imaging (MRI) was not performed. Brown syndrome and monocular elevation deficiency were considered in the preoperative differential diagnosis, and surgery was planned with intraoperative forced duction testing and direct exploration to define the mechanical component of the restriction.

Under general anesthesia, multidirectional forced duction testing was performed, including exaggerated traction testing^[^16^]^. There was no appreciable restriction during passive elevation in adduction in the superonasal position of the left eye. The greatest mechanical resistance was encountered at a focal central point in the superior plane. Passive infraduction and horizontal rotations were unrestricted. When the elevated globe was moved from nasal to temporal positions across the superior plane, it appeared to pass over the focal central point of resistance, giving a bridge-like impression. Numerical directional forced duction grades were not recorded.

Superior exploration initially demonstrated a normal-appearing superior rectus muscle with a small focal tissue attachment at its nasal aspect (Fig. 2A). This attachment was released; however, Repeat forced duction testing continued to demonstrate substantial mechanical restriction during passive elevation. Further posterior exploration was therefore undertaken using a small muscle hook. At the retro-equatorial level, a deep band-like tissue was identified centrally within the superior/posterior operative field (Fig. 2B).

**Figure 2.**
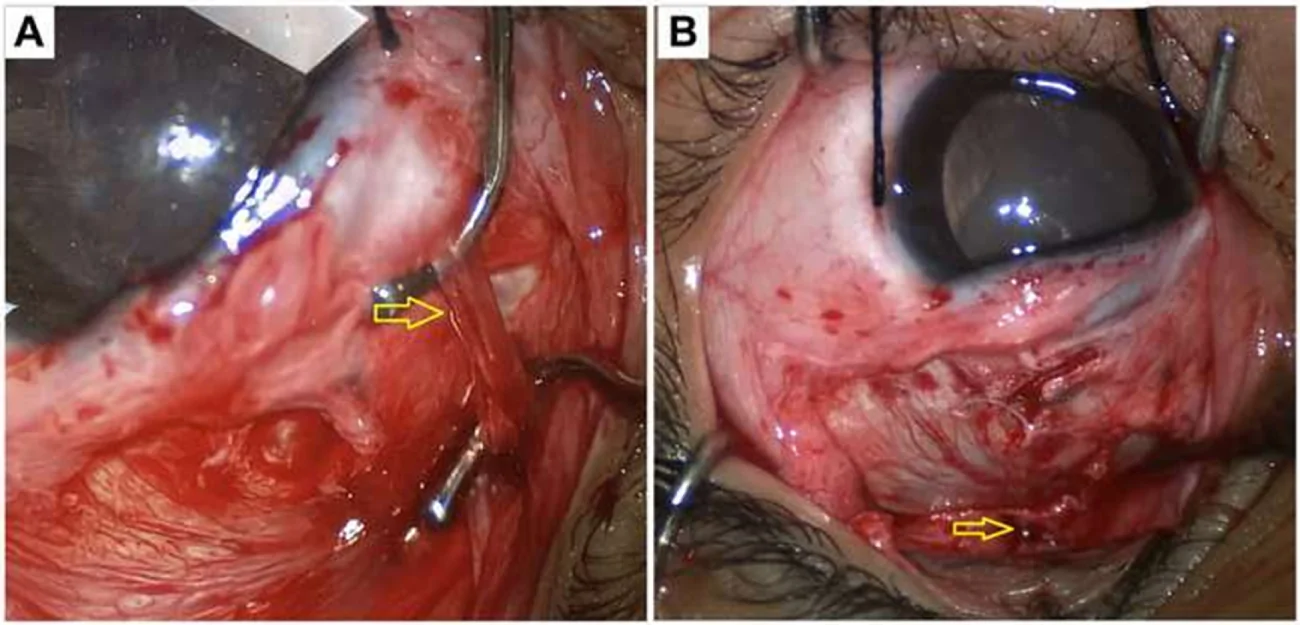
Intraoperative findings: (A) Focal abnormal tissue at the nasal aspect of the superior rectus region, indicated by the yellow arrow. (B) Deep retro-equatorial band in the superior-posterior operative field, indicated by the yellow arrow, with a muscle hook adjacent to or beneath it. No tissue from either abnormal area was submitted for histopathological examination. The images do not establish the identity of a specific muscle, tendon, or fibrotic tissue, nor do they demonstrate anatomical continuity between the two findings.

The deep retro-equatorial tissue was released progressively as far posteriorly as was safely possible. Serial forced duction testing demonstrated marked improvement in passive elevation, although mild residual restriction remained in the superior plane. Passive infraduction and horizontal rotations remained unrestricted. Further posterior dissection was not undertaken because the tissue extended deeper into the posterior orbit, where additional exploration could have endangered neurovascular structures or the optic nerve. No specimen was submitted for histopathological examination from either the focal attachment in the superior rectus-region or the deeper retro-equatorial tissue; therefore, their precise histological identities and anatomical origins could not be established.

The inferior rectus was not explored because passive infraduction was unrestricted and no mechanical resistance was identified in the inferior plane. In the absence of a demonstrable mechanical target inferiorly, additional inferior rectus exploration was considered unnecessary and would have entailed further operative dissection. The anatomy of the inferior rectus was therefore not directly verified.

A 10 mm recession of the left lateral rectus was subsequently performed to address the 20 PD exotropia. The horizontal deviation was managed unilaterally in the affected eye, rather than operating on the fellow eye.

Postoperative recovery was uneventful. At 6 months, there was a small flick esotropia with 14 PD of residual right hypotropia, equivalent to left hypertropia.

At the 12 month examination, horizontal alignment was orthotropic, with no residual exotropia or esotropia. Residual right hypotropia measured 12 PD at distance and approximately 8–10 PD at near. Left supraduction and infraduction had each improved from −3 preoperatively to approximately −2, while abduction and adduction remained full (Fig. 3). The vertical deviation, therefore, remained in the same direction throughout follow-up, decreasing from 40 PD preoperatively to 14 PD at 6 months and to approximately 8–12 PD at 12 months. A formal patient or guardian perspective was not collected.

**Figure 3.**
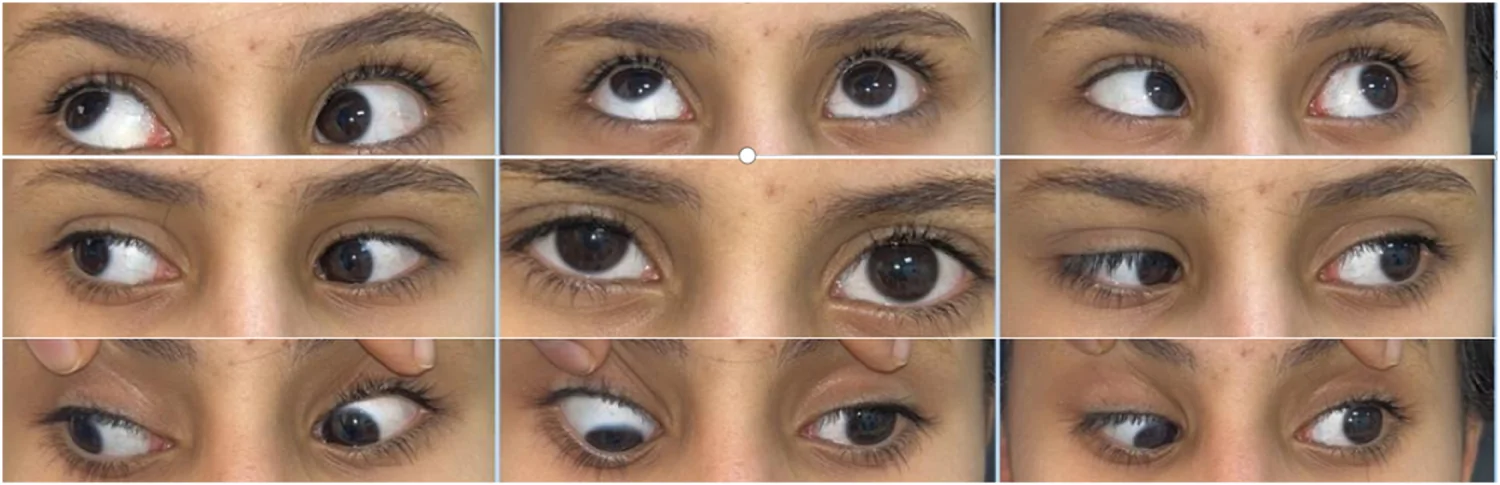
Postoperative nine-gaze ocular-motility photographs at 12 months. Clinical examination documented horizontal orthotropia and residual right hypotropia measuring 12 PD at distance and approximately 8–10 PD at near. Left supraduction and infraduction were each limited by approximately −2, while abduction and adduction remained full.

## Clinical discussion

The principal observation in this case was the marked reduction in passive restriction after stepwise release of a deep retro-equatorial band encountered in the superior-posterior orbit. Serial forced duction testing demonstrated a clear improvement in passive elevation after release, supporting an important mechanical component of the motility disorder. However, the deep tissue could not be completely released because it extended posteriorly into an area where further dissection could have endangered the orbital neurovascular structures and optic nerve. Mild residual restriction of passive elevation therefore remained at the completion of surgery. The operative findings should consequently not be interpreted as demonstrating complete elimination of the restrictive process or as establishing that the deep band alone accounted for the entire clinical phenotype. A separate focal tissue attachment was also encountered in the superior rectus region, and the anatomical relationship and relative functional contribution of the two findings could not be determined.

## Differential diagnosis and imaging

Mechanical and anatomical causes of congenital restrictive strabismus include anomalous accessory muscle or tissue bands^[^2–5^]^, extraocular muscle or tendon dysplasia^[^1,2^]^, and congenital orbital fibrosis or fibrosis-like abnormalities^[^6–10^]^. Reported abnormalities include anomalous tissue adjacent to the rectus muscles, abnormal superior rectus/superior oblique complexes^[^1,2^]^, and deeper accessory orbital structures associated with complex incomitant deviations^[^4,5^]^. The focal superior rectus-region attachment and deeper retro-equatorial band in this patient may therefore represent separate abnormalities or components of a broader developmental orbital anomaly.

Brown syndrome and monocular elevation deficiency were considered preoperatively. Intraoperative forced duction testing demonstrated no appreciable restriction during passive elevation in adduction in the superonasal position of the left eye. Instead, the greatest resistance was localized to a focal central point in the superior plane, while passive infraduction and horizontal rotations were unrestricted. This distribution was not characteristic of an isolated classic Brown syndrome, in which restriction of elevation in adduction would be expected. Serial forced duction testing and direct exploration were therefore used to define the mechanical component rather than relying solely on the preoperative diagnostic label.

Congenital cranial dysinnervation was also considered in the differential diagnosis. Importantly, the apparent right upper-eyelid asymmetry was formally evaluated by the oculoplastic service and diagnosed as pseudoptosis associated with the right hypotropia rather than true congenital ptosis. MRD-1 was 3 mm in both eyes when each eye was fixating, levator function was greater than 14 mm bilaterally, and there was no lid lag or lagophthalmos. The eyelid appearance should therefore not be interpreted as evidence of the congenital ptosis typically associated with classic CFEOM. Together with the absence of profound bilateral ophthalmoplegia, these findings make the classic phenotypes of CFEOM1 and CFEOM2 considerably less likely.

CFEOM3, however, cannot be completely excluded solely on the basis of pseudoptosis and preserved levator function because it may be unilateral, asymmetric, and variably expressed^[^13,14^]^. Dedicated orbital and cranial nerve MRI and, when clinically indicated, genetic assessment would be required to investigate a congenital cranial dysinnervation disorder more definitively. Identification of a mechanical tether does not exclude coexisting extraocular muscle dysplasia or dysinnervation.

The congenital onset preceding the minor trauma, stable clinical course, and absence of inflammatory disease or previous orbital surgery make an acquired restrictive process less likely. Nevertheless, intact orbital walls on CT do not exclude congenital soft-tissue abnormalities. Dedicated orbital MRI could have provided additional information regarding extraocular muscle morphology and course, tendons, accessory structures, and the relationship of the abnormal tissue to deeper orbital structures^[^5,7,8,14^]^. Cranial nerve imaging could also have contributed to the assessment for congenital dysinnervation^[^14^]^. The absence of MRI, therefore, remains an important diagnostic limitation.

## Postoperative alignment and residual motility

At 6 months, the patient had a small flick esotropia with 14 PD of residual right hypotropia, which was equivalent to left hypertropia. At the 12 month examination, horizontal alignment was orthotropic, with no residual exotropia or esotropia. Residual right hypotropia measured 12 PD at distance and approximately 8–10 PD at near. The vertical deviation therefore decreased substantially from 40 PD preoperatively to 14 PD at 6 months and approximately 8–12 PD at 12 months, while remaining in the same direction throughout follow-up.

The active motility findings are particularly relevant to the interpretation of the surgical result. Preoperatively, both left supraduction and infraduction were limited to −3. At 12 months, both had improved to approximately −2, while abduction and adduction remained full. Thus, both vertical ductions improved, but neither returned to normal.

This residual active vertical limitation is consistent with the operative finding that the restrictive process could not be completely released. The deep retro-equatorial tissue extended posteriorly beyond the safely accessible operative field, and further dissection was deliberately stopped because of the potential risk to the optic nerve and adjacent neurovascular structures. Serial forced duction testing demonstrated marked improvement in passive elevation after release of the accessible portion of the band, but mild residual superior restriction remained. The improvement from −3 to approximately −2 in both supraduction and infraduction at 12 months should therefore be interpreted as partial functional improvement following an intentionally incomplete release performed for anatomical safety, rather than as complete restoration of normal motility.

Nevertheless, the remaining limitation of active vertical ductions cannot be attributed solely to the unreleased posterior portion of the band. Active motility and passive forced duction findings represent different physiological processes. Residual active limitation may reflect the remaining mechanical restriction, associated extraocular muscle dysplasia, congenital dysinnervation, altered postoperative orbital biomechanics, or a combination of these factors. The available clinical and operative findings do not permit these mechanisms to be separated definitively.

The inferior rectus was not explored because passive infraduction was unrestricted during intraoperative forced duction testing, and no resistance was identified in the inferior plane. Additional inferior rectus exploration would, therefore, have required further dissection without a defined mechanical target. Although the inferior rectus anatomy was not directly verified, active infraduction improved from −3 preoperatively to approximately −2 at 12 months. This distinction is important: the preoperative infraduction deficit represented an active motility abnormality, whereas intraoperative passive infraduction was unrestricted.

The 10 mm left lateral rectus recession was selected to address the measured 20 PD exotropia through a unilateral procedure in the affected eye, avoiding horizontal muscle surgery in the fellow eye. Unilateral lateral rectus recession is an established single-muscle procedure for small-to-moderate exotropia; recessions of 7–10 mm have been reported for deviations of 15–35 PD^[^17^]^, and a uniform 10 mm recession has been used for exotropia of up to 25 PD without postoperative limitation of abduction^[^18^]^. In intermittent exotropia of 20 PD, larger recessions within the 8.0–9.0 mm range were associated with numerically lower recurrence within 2 years^[^19^]^. The selected recession was therefore at the upper end but within the published range for this magnitude of exotropia. Horizontal alignment was orthotropic at 12 months.

However, these published series primarily involved intermittent or other comitant exotropia rather than congenital restrictive strabismus^[^17–20^]^. The horizontal result, therefore, cannot be generalized to similar restrictive cases. In addition, recurrence after unilateral lateral rectus recession may occur beyond the first postoperative year^[^18^]^, so longer follow-up would be required to establish durability. The horizontal result also cannot be attributed to the lateral rectus recession alone because the release of abnormal orbital tissue may have altered globe mechanics and the balance of extraocular forces.

Overall, this case demonstrates that serial forced duction testing can help localize and assess the response to an unexpected mechanical restriction during surgery. The improvement of both supraduction and infraduction from −3 preoperatively to approximately −2 at 12 months occurred despite intentionally leaving a deeply extending portion of the restrictive tissue undisturbed because of the risk of optic nerve and neurovascular injury. The principal surgical objective was therefore not complete excision of all abnormal tissue, but rather maximal safe release of the accessible restriction while avoiding potentially serious orbital complications.

## Limitations

This report is limited by the absence of dedicated orbital MRI, genetic assessment, and histopathological confirmation of the abnormal tissues encountered intraoperatively. The precise tissue identity, full posterior course of the deep retro-equatorial band, and its anatomical relationship to the superior rectus-region finding therefore remain unverified. Posterior dissection was intentionally limited because of the potential risk to deep orbital neurovascular structures and the optic nerve, and a portion of the deeply extending restrictive tissue could not be safely released. The inferior rectus was not explored because passive infraduction was unrestricted intraoperatively; its anatomy therefore remains unverified.

Forced duction testing was qualitative. The restriction was localized to passive elevation at a focal central point in the superior plane; no appreciable restriction was present in the superonasal position during elevation in adduction, while passive infraduction and horizontal rotations were unrestricted. Numerical directional forced duction grades were not recorded. The absence of quantitative forced duction scores limits reproducibility and direct numerical comparison with previously reported cases of anomalous orbital bands. Active duction grades cannot substitute for quantitative passive forced duction measurements.

Finally, this is a single case involving both the release of abnormal orbital tissue and unilateral lateral rectus recession. The relative contribution of each intervention to the postoperative alignment cannot be isolated, and the findings cannot establish lesion-specific causality or predict surgical outcomes in other patients. Follow-up was limited to 12 months, and a later recurrence of exotropia, which has been reported after unilateral lateral rectus recession^[^18^]^, cannot be excluded.

## Conclusion

Stepwise release of a deep retro-equatorial band was followed by marked improvement in passive elevation in a child with congenital unilateral restrictive strabismus, although mild residual superior restriction remained because further posterior dissection was limited by the risk to the deep orbital neurovascular structures and the optic nerve. At 12 months, right hypotropia had decreased from 40 PD preoperatively to 12 PD at distance and approximately 8–10 PD at near, while horizontal alignment was orthotropic. Both left supraduction and infraduction improved from −3 preoperatively to approximately −2 at final follow-up, while abduction and adduction remained full. These findings support an important mechanical contribution to the motility disorder, with substantial but incomplete improvement in vertical alignment and ocular motility. They do not establish the precise identity of the abnormal tissue, nor do they exclude coexisting extraocular muscle dysplasia or congenital dysinnervation. Serial forced duction testing may help guide the targeted release of abnormal orbital tissue, while deep posterior exploration should remain limited by anatomical safety considerations.

## Data Availability

All data relevant to the case are included in the article. Further information is available from the corresponding author on reasonable request, subject to patient privacy and institutional requirements.
